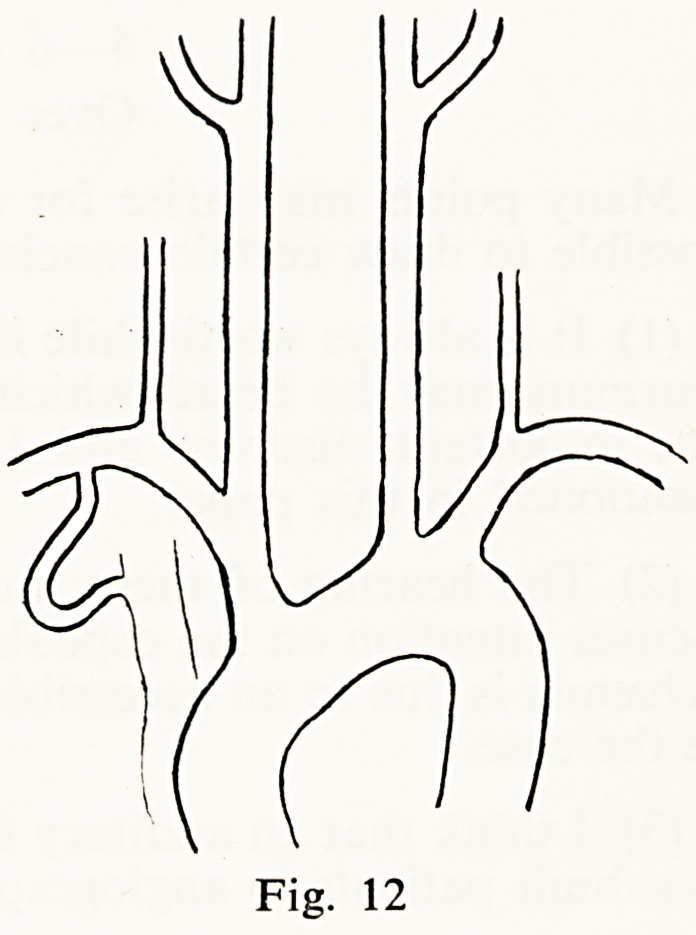# On Listening to the Neck

**Published:** 1967-01

**Authors:** T. W. Lloyd

**Affiliations:** The Royal Hospital, Gloucester


### ON LISTENING TO THE NECK

BY

T. W. LLOYD, D.M., M.R.C.P.

*The Royal Hospital, Gloucester.*

The real importance of listening for murmurs in the neck was borne in upon me as the result of seeing two cases in quick succession in the summer of 1965.

A man of 63 was referred by his solicitors shortly before standing trial for causing the death of a woman by dangerous driving. The only defence was that a witness had seen him sitting upright at the wheel making no attempt to avoid a totally unnecessary collision. A strictly routine examination would have revealed only moderate hypertension (190/94) in the right arm; but there was a murmur above the left clavicle and reduced pulse pressure (130/94) in the left arm, and this discovery led to the demonstration of stenosis of the left subclavian artery (Fig. 1) with reversed flow of blood down the left vertebral artery (Fig. 2), the " subclavian steal " syndrome. Evidence of this disorder secured his acquittal. This fascinating condition has recently been comprehensively reviewed by Patel and Toole (1965).

An Italian woman aged 31 had suffered continuous obstinate infection of the urine for a year, with pain in the left loin. This proved to be due to a renal stone which was later removed. The initial examination revealed a right brachial blood pressure of 180/90, and the cause might have been accepted as pyelonephritis but for the discovery of a murmur above the left clavicle. This led to the demonstration of a complete obstruction of the left subclavian artery, with irregularity and indeed coarctation of the aorta above the cœliac axis (Fig. 3). This mid-aortic syndrome (Sen *et al*, 1963), due to diffuse aortitis, has a very different prognosis from renal stone. In this case, which presents none of the systemic symptoms often encountered in diffuse aortitis (Schrire and Asherson 1964, Strachan 1964), the prognosis may depend largely on the reaction of the left ventricle to the aortic coarctation.

These impressive cases led to the inclusion of auscultation over the subclavian, carotid and vertebral arteries in the routine examination of all new patients referred to my unit, and in six months 27 more patients were found to have focal asymmetrical murmurs in the neck, an incidence of more than 5 per cent and actually more than one a week. None of these were associated with aortic valve bruit. Some were soft, some continuous, but most were harsh ejection murmurs lasting through systole.

The 29 patients on which this paper is based include the case of diffuse aortitis already mentioned and one man of 67 who had a small aneurysm of the subclavian artery distal to a cervical rib. 4 young patients had murmurs over the lower part of the right external jugular vein, the " bruit de diable ", a condition which can be readily recognised for the continuous murmur ceases abruptly when a finger is laid on the jugular vein higher in the neck. Two of these were young patients in their 'teens with severe anæmia, but the sound has no devilish significance.

In 23 patients atherosclerosis was thought to cause the murmur. All the 14 male patients were under the age of 68 and all but 3 were over 60. The average age of the women was higher and 2 patients were over 80. In 4 of these arteriosclerotic patients there were no symptoms and the finding was quite unexpected. The remaining 19 had symptoms suggesting transient cerebral ischæmia or actual stroke. These are listed in order of frequency in the Tables. Their importance is that often they had occurred over an extended period during which full assessment of the case would have been possible if the need had been recognised; if indeed the murmur had been heard. For instance, a woman of 58 had transient weakness of the left arm twice in 3 months. A harsh murmur was heard over the right carotid sinus and angiography demonstrated a thrombus obstructing the internal carotid artery (Fig. 4). Successful surgery in this case must surely have greatly improved her prognosis and no further symptoms have occurred in the first 6 months after endarterectomy.

A man of 62 suffered giddy turns with transient ataxia for 3 years and had loud murmurs on the left side of the neck. His arch aortogram (Fig. 5) showed gross disease of the left subclavian, severe stenosis of the left internal carotid and complete obstruction of the right internal carotid artery. Despite the extensive disease a plaque of atheroma was removed from the left internal carotid with complete relief of his symptoms.

The murmurs we heard were for the most part persistent but this is not an essential feature of the significant bruit.

A man of 36 had a right hemiparesis which disappeared almost completely in 3 days. The only probable cause found was a tortuous internal carotid (Fig. 6) and in such cases one may hear a murmur only with the head in certain positions.

A woman of 53 developed pain, weakness, and numbness of her right arm over a period of 3 weeks followed by slowly increasing weakness of the right leg. There were loud murmurs over each carotid sinus. A left carotid angiogram showed a typical stenosis of the first part of the internal carotid. At a later stage the corresponding murmur was no longer present and arch aortography showed complete obstruction of the left carotid with a tight stenosis of the right internal carotid where a murmur persisted.

A variable murmur may therefore indicate a variable degree of obstruction, yet still have been significant when heard; and a murmur may be significant of stenosis yet the stenosis producing it may not be the one which matters.

A man had 3 blackouts, the last continuing for more than an hour. He had murmurs over the right carotid sinus and the left vertebral artery. Arch aortography (Fig. 7) showed that while the latter was due to a cuff-like stenosis of the vertebral orifice the former was caused by a stenosis of the external carotid artery, not the internal, and was presumably not relevant. His subsequent progress was characterised by recurrent episodes of vertigo, certainly due to brain stem ischæmia, which responded in some measure to wearing a cervical splint.

Another man aged 63 had visual disturbance and instability of gait with hemiparæsthesiæ for one week, and this led to an examination at which a murmur was demonstrated over the left carotid sinus. It was very localised and not very loud. Carotid angiography showed a moderate narrowing of the first centimetre of the internal carotid; but the serious matter was a very marked stenosis of the same vessel in the syphon (Fig. 8). Complete right hemiplegia developed after this examination.

In this case (and indeed it seems to be a common phenomenon) when the orifice of one internal carotid artery was affected enough to produce a murmur, there was a similar change of less degree in the opposite internal carotid even

Fig. 1

Fig. 2

Fig. 3

Fig. 4

Fig. 5

Fig. 6

though no murmur was present. The absence of a murmur therefore may mean that there is no narrowing, slight narrowing, complete obstruction, or flow through unusual channels.

In this context another case was of considerable interest. A man of 46 had a sudden stroke with left hemiparesis. A loud harsh murmur was heard over the right carotid sinus only. He made a fairly good recovery but 6 months later, when the carotid murmur had vanished, a loud murmur was heard over the right subclavian. Arch aortogram now showed complete obliteration of the subclavian with filling of the axillary artery through collaterals (Fig. 9), so good a collateral flow that there was no considerable fall in pulse pressure.

It is worth remembering obstruction of the subclavian when considering pain in the arm. The patient with subclavian steal quoted above still suffers quite severe intermittent claudication in the left arm, and recently I have seen another patient in whom it is not altogether clear whether the pain he feels in the left arm is due to angina pectoris or to subclavian ischæmia. Pain in the arm may also be due to embolic episodes, particularly associated with cervical rib and subclavian aneurysm (Gunning *et al*, 1964).

Atheroma can progress to a very advanced stage without producing actual symptoms and extraordinary degrees of obstruction to the cephalad blood flow occur without causing actual death.

A man of 63 worked in the locomotive sheds at Gloucester. I saw him after he had a transient left hemiparesis and noted loud murmurs in the neck, and absent radial pulses. He subsequently suffered a complete left hemiplegia and aortography showed a remarkable picture (Fig. 10)—a severe coarctation of the innominate with complete obstruction of both common carotids, severe narrowing of the left subclavian and cuff-like stenosis at the origin of the left vertebral artery. With all this he was thought well enough for a surgical by-pass operation to be attempted.

A woman of 64 who had suffered weakness of her left arm for 3 months was admitted with complete left hemiplegia and died 10 days later of thrombosis of the superior mesenteric artery. Radiography of the dissected aortic arch showed a flow of medium up the left common carotid only and there were numerous thrombi, old and recent, in the vessels, yet she had been able to feel and express the distress she experienced from infarction of the bowel with only this cephalad blood flow (Fig. 11).

Possibly the study of cases such as these will remind us that stenosis *per se* may be of little importance. Brice, Dowsett and Lowe (1964) have shown that normal carotid arteries can be clamped down to a tiny fraction of their normal cross section without causing a fall in blood pressure on the distal side or any diminution in flow along the vessel; these factors are decided by the peripheral vascular resistance and the cardiac output. The latter is particularly important, and the choice of preoperative sedative in carotid angiography may be of critical significance in avoiding ischæmic disaster due to hypotension.

The real significance of stenosis of course is the threat of a thrombotic complication at the site, but often we have to ignore this. For instance in a case already quoted (Fig. 4) the blood flow past the obstruction served only the right middle cerebral field. The whole of the left carotid field and the right anterior cerebral artery was adequately supplied through the left internal carotid, but this was itself moderately narrowed at its orifice. It is lamentably necessary to remember that we are dealing with incidents in a widespread and

Fig. 7

Fig. 8

Fig. 9

Fig. 10

Fig. 11

Fig. 12

progressive disease process. We are attempting only to preserve function while that is possible.

There are other possible causes of murmur in the neck. Apart from soft bruits over the gland, thrilling, torrential sounds are sometimes heard in association with toxic goitre, sounds æsthetically satisfying as well as clinically useful. Occasionally in a person with a thin neck it is possible to produce a carotid murmur accidentally by pressure of the stethoscope bell. And other oddities occur. During this same period a very exceptional patient was seen by Dr. R. F. Jarrett, a woman of 50 having a congenital arterio-venous shunt between the thyreo-cervical trunk and the superior vena cava (Fig. 12). Attention had been drawn to this by hearing a loud murmur above the right clavicle. The patient had suffered dizzy turns, faintness, and a variety of symptoms which had been thought to be hypochondriacal.

### TABLE I

*The frequency of cerebal ischæmic symptoms experienced by patients with murmurs in the neck:—*

| | |
|---|---|
| Dizzy Spells ... ... | 6 |
| Transient paresis ... | 6 |
| Visual disturbance ... | 5 |
| Drop seizures ... ... | 3 |
| Headache ... ... | 3 |
| Mental slowing ... | 3 |
| Blackout ... ... | 2 |
| Ataxia ... ... | 2 |

*The duration of these symptoms before diagnosis or major stroke, whichever occurred first was:—*

| | |
|---|---|
| 1 week ... ... ... | 5 |
| 2—4 weeks ... ... | 3 |
| 2—4 months ... ... | 5 |
| 4—6 months ... ... | 4 |
| Over 6 months ... | 2 |

Many points may arise for discussion out of this series of cases, but it is possible to draw certain conclusions.

(1) It is always worthwhile listening to the neck. In a small number of cases murmurs may be heard which will direct attention to a totally unsuspected but important disorder and I would remind you of the first two cases I mentioned in this paper.

(2) The hearing of these murmurs in cases of stroke, or stuttering stroke, focuses attention on the cephalad blood flow and raises the possibility that the ischæmia is due to an accessible stenosis, even though this may not necessarily be the case.

(3) I think that an auditory stimulus of this kind has increased my readiness to submit patients to angiography when the history of transient weakness of a limb or visual disturbance might not have been sufficient and I am sure that if general practitioners of today and of the future are taught to listen to the neck more opportunities for preventive surgery will be found, as well as a great many cases that will be very difficult to assess.

### REFERENCES

Brice, J. G., Dowsett, D. J. and Lowe, R. D. (1964), *B.M.J.*, **ii**, 1363.

Patel, A. and Toole, J. F. (1965), *Medicine* **44**, 289.

Gunning, A. J., Pickering, G. W., Robb-Smith, A. H. T., Ross Russell R. (1964), *Quart. J. Med.*, **33**, 133.

Schrire, V. and Asherson, R. A. (1964), *ibid.*, **33**, 439.

Strachan, R. (1964), *ibid.*, **33**, 57.

## Figures and Tables

**Fig. 1 f1:**
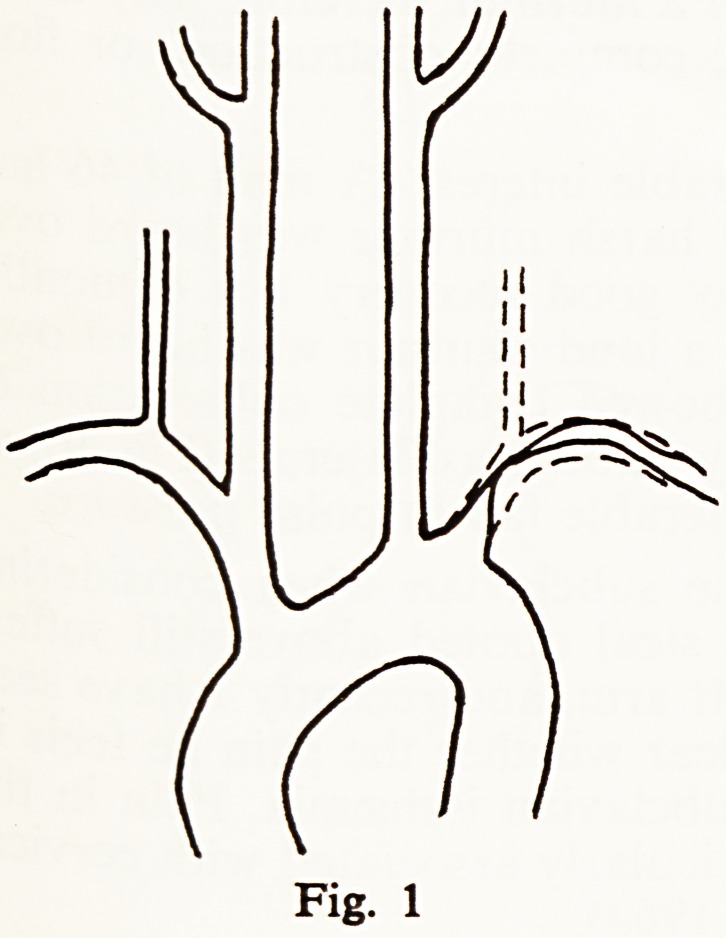


**Fig. 2 f2:**
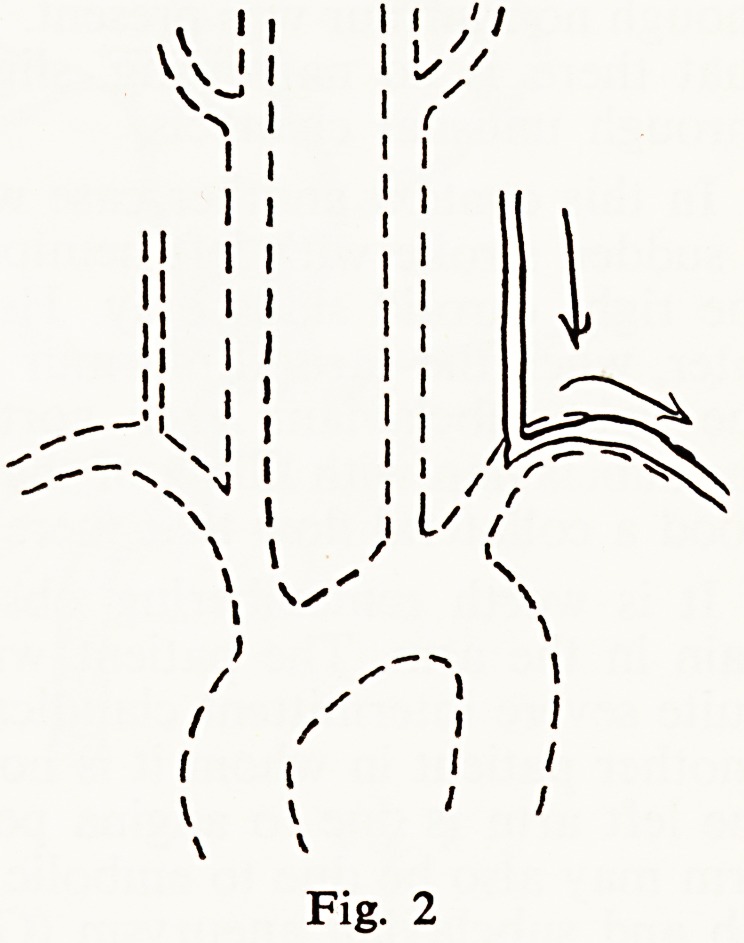


**Fig. 3 f3:**
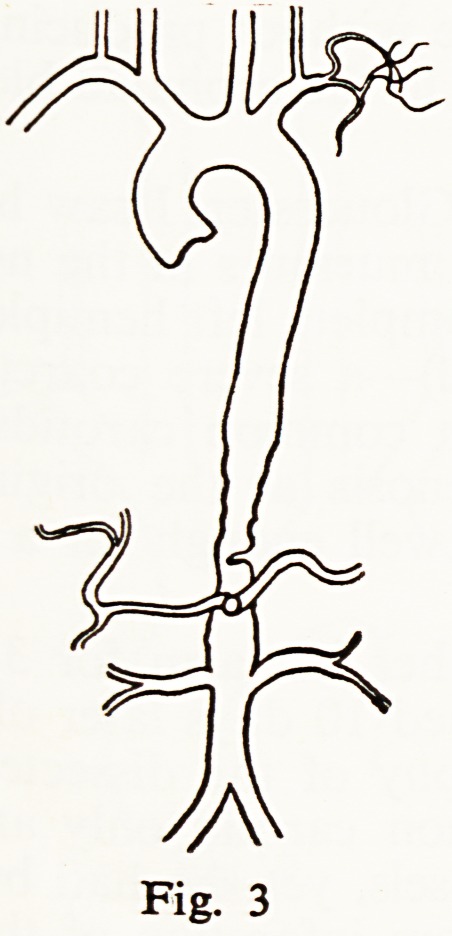


**Fig. 4 f4:**
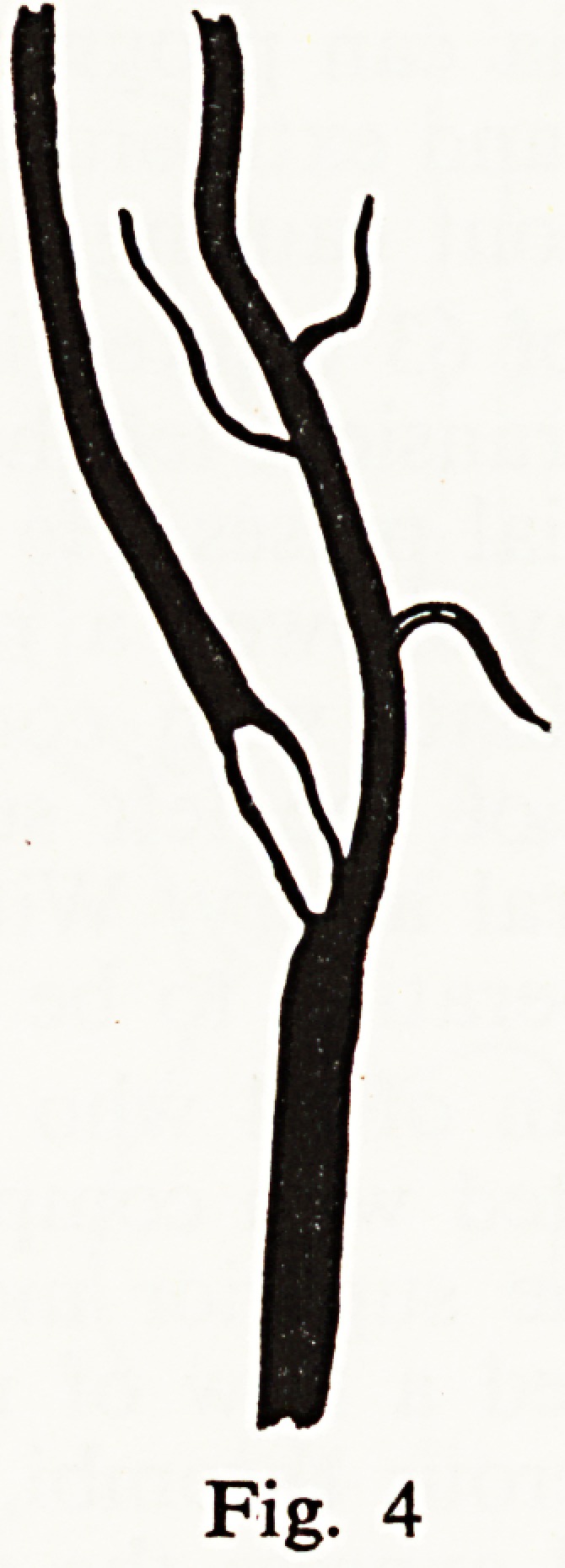


**Fig. 5 f5:**
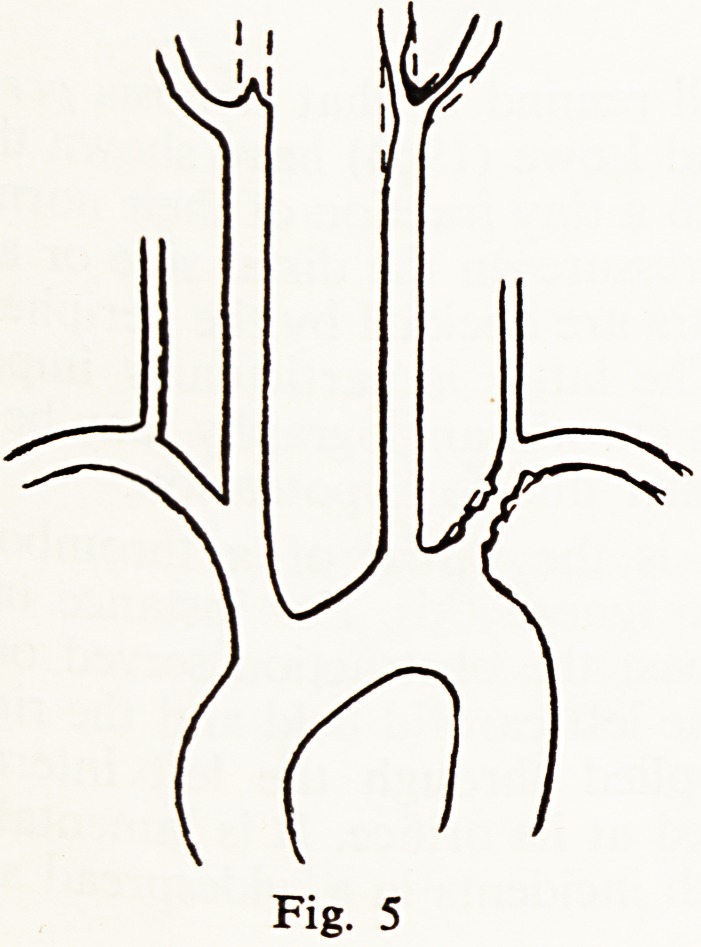


**Fig. 6 f6:**
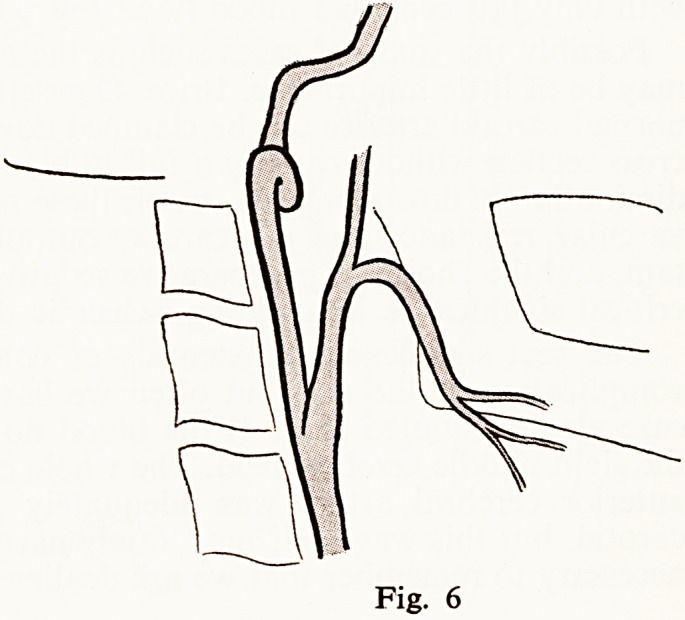


**Fig. 7 f7:**
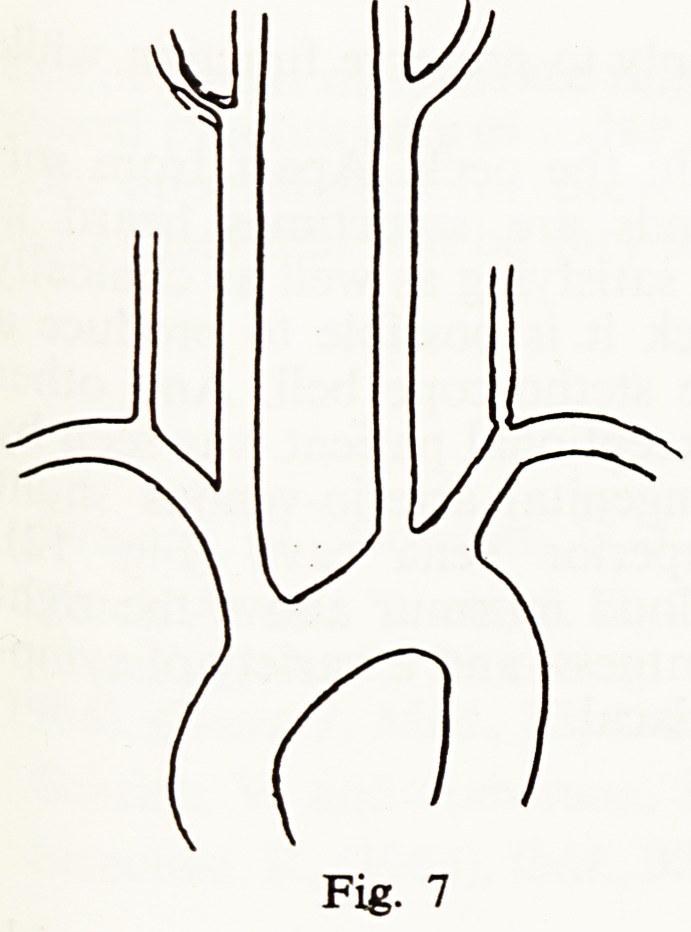


**Fig. 8 f8:**
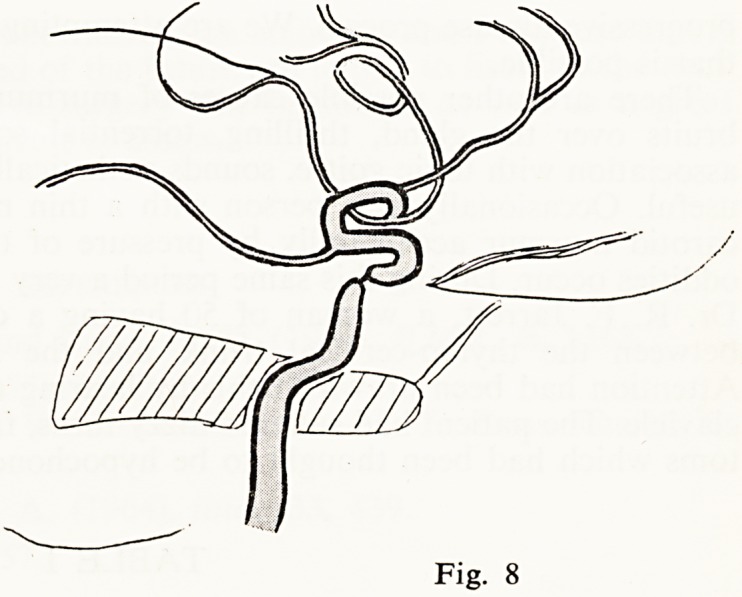


**Fig. 9 f9:**
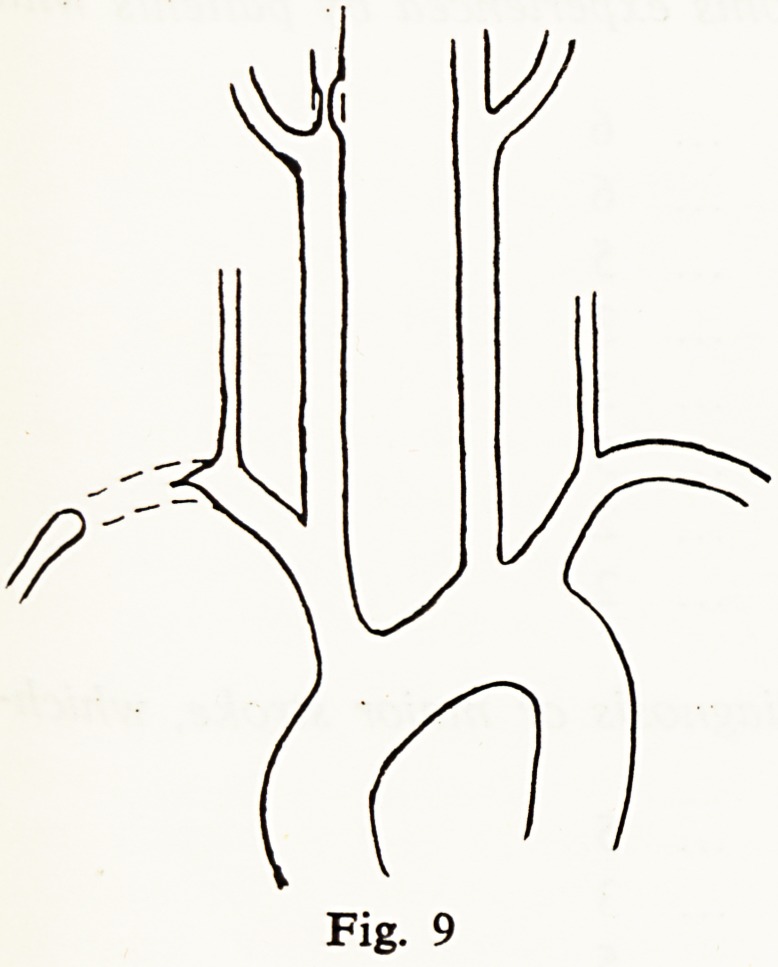


**Fig. 10 f10:**
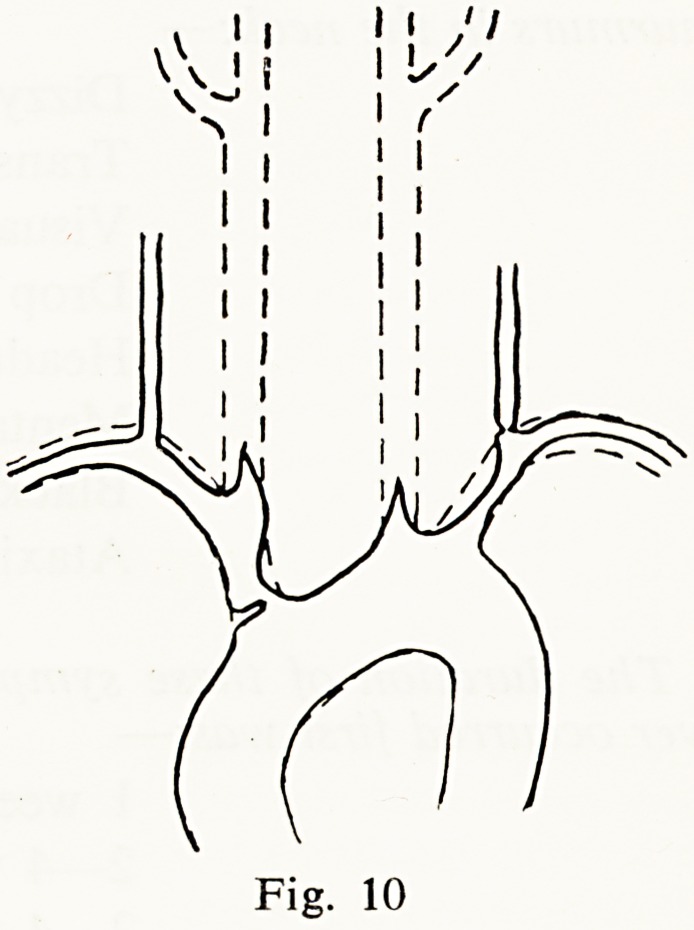


**Fig. 11 f11:**
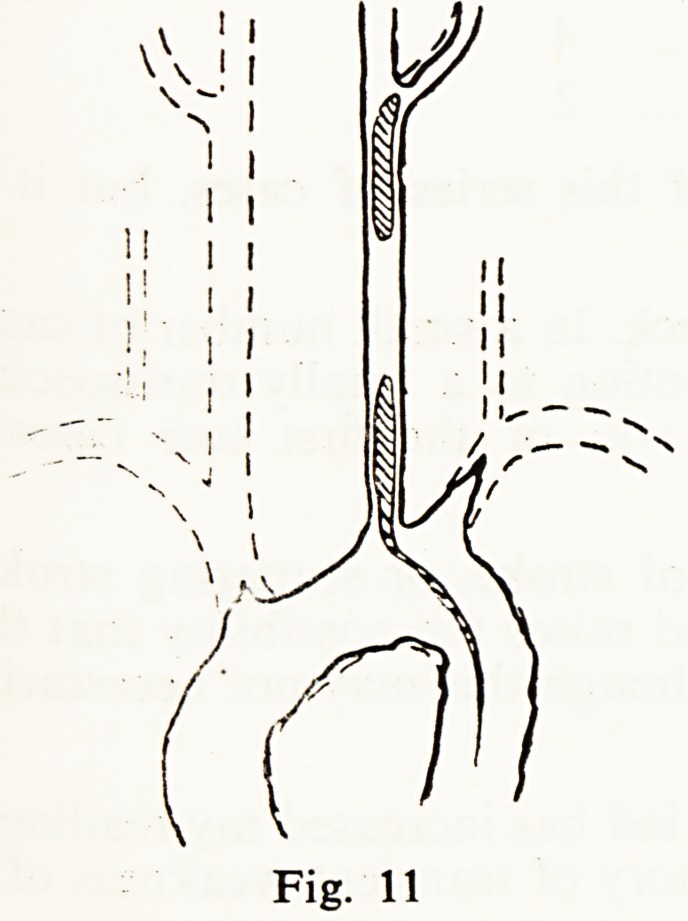


**Fig. 12 f12:**